# Investigating the action model of the resistance enhancement induced by bacterial volatile organic compounds against *Botrytis cinerea* in tomato fruit

**DOI:** 10.3389/fpls.2024.1475416

**Published:** 2024-11-26

**Authors:** Jianhua Chen, Kexin Cao, Xuan Lu, Ding Huang, Ruhong Ming, Rumei Lu, Rongshao Huang, Liangbo Li

**Affiliations:** ^1^ College of Pharmacy, Guangxi University of Chinese Medicine, Nanning, China; ^2^ College of Agriculture, Guangxi University, Nanning, China

**Keywords:** MVOCs volatile organic compounds (VOCs), tomato, grey mould, phenolic acids, disease resistance

## Abstract

**Introduction:**

Inducing natural resistance against pathogen infection in postharvest tomatoes is a sustainable strategy for reducing postharvest losses. The action model underlying the resistance enhancement of tomatoes induced by bacterial volatile organic compounds (VOCs) against *Botrytis cinerea*, however, have not been explored.

**Methods:**

In this study, RNA-seq, metabolomics and physiological analysis were used to evaluate global change of defense response induced by VOCs in tomatoes.

**Results:**

The application of VOCs inhibited the damage to tomatoes caused by *B. cinerea*. VOCs treatment had remarkable beneficial effects on the activities of the main defence-related enzymes, including chitinases, glucanases, peroxidases, ascorbate peroxidases, polyphenol oxidases, and phenylalanine ammonia-lyases. The expression of response genes involved in salicylic acid and jasmonic acid biosynthesis and signalling pathways was enhanced upon VOCs treatment. Metabolomics data demonstrated that VOC treatment triggered the accumulation of phenolic acids, including substrates in phenolic acid biosynthesis pathways, hydroxycinnamic acid, hydroxybenzoic acid, and their derivatives. Transcriptomics analysis and qRT-PCR verification revealed that VOCs treatment significantly upregulates the expression of core genes related to phenolic acid biosynthesis, specifically in shikimate pathway (*SlDAHPS*, *SlSDH*, *SlCS*, and *SlADT3*) and phenylalanine metabolic pathway (*SlPAL*, *Sl4CL*, *SlBAHD1*, *SlCYP98A2* and *SlCAP84A1*).

**Discussion:**

Results confirmed that VOCs enhanced tomatoes postharvest resistance against *B. cinerea* by regulating defence enzyme activity, SA/JA signalling, and phenolic acid biosynthesis pathway. This study provides new insights into the mechanisms by which VOCs fumigation manages postharvest grey mould in tomatoes.

## 1 Introduction

Grey mould, also known as *Botrytis cinerea*, is a widely distributed fungal pathogen that causes significant damage to crops before and after harvest (Rahman et al., 2024). It is considered one of the most important plant pathogens in terms of economic impact and estimated to cause economic losses ranging from 10 to 100 billion U.S. dollars worldwide (Li et al., 2018). In postharvest situations, the fungus can cause storage rot, rendering crops unsuitable for sale or consumption. As a necrotrophic fungus, its main characteristic is the ability to induce host cell death to secure its nutrient supply (Wang et al., 2024), which is primarily caused by the widespread secretion of non-selective toxins and plant cell wall-degrading enzymes (van Kan, 2006). Recent studies have revealed that *B. cinerea* possesses a more diversified toolbox than previously believed (Bi et al., 2023; Cheung et al., 2020). Controlling the spread of *B. cinerea*, like many other fungal pathogens, primarily relies on chemical intervention. In fact, a significant portion, around 8% of the worldwide fungicide market is dedicated to managing this specific pathogen (Nishimoto, 2019). However, the extensive use of chemical fungicides causes many problems, especially environmental pollution and hazardous effects on human health (Jankowska et al., 2016). Consequently, there is an urgent need to identify alternative toxic-free and eco-friendly methods to extend the shelf life of postharvest crops and meet the stringent requirements of environmental safety and public acceptance.

Induced natural resistance in postharvest fruits and vegetables using biotic or abiotic elicitors has become an attractive and sustainable strategy for controlling disease and managing decay (Prusky and Romanazzi, 2023). The state of induced resistance is characterised by the activation of latent defence mechanisms with higher expression during subsequent attacks by pathogens. These mechanisms include physiological and biochemical changes and their molecular responses (generation of ROS and activation of the antioxidant system, production of pathogenesis-related (PR) proteins, MAPK signalling and accumulation of phytoalexins) and synthesis of structural barriers (callose, glycoproteins, phenols, ect.) in the host to strength the cellular structure (Romanazzi et al., 2016; Dukare et al., 2018; Thankappan et al., 2022). Meanwhile, abundant evidence suggested that plant synthesis of some hormones, such as salicylic acid (SA), jasmonic acid (JA) and ethylene (ET), along with their intricate signal transduction networks, served as prerequisites for inducing postharvest crops’ resistance against pathogens (Singh et al., 2021). Understanding the effects and efficiency of defence activation mediated by biotic or abiotic stimulators requires characterization of the changes in plant defence patterns and mechanisms during the interaction between hosts, inducers, and pathogens.

Microorganisms, primarily fungi, bacteria, and yeast, can produce a wide range of microbial volatile organic compounds (VOCs) characterised by low boiling points and low molecular weights (Macías-Rubalcava et al., 2018). Due to its excellent antifungal properties, it has been gradually used as natural alternatives to artificial fumigants to control various postharvest diseases (Mari et al., 2016; Zhong et al., 2021). Moreover, bacteria-produced volatile organic compounds also have been reported as a potent elicitor of plant defence responses against certain plant fungi (Romanazzi et al., 2016; Farag et al., 2013; Zhou et al., 2019). For instance, Ryu et al. (2004) reported that bacterial volatiles emitted from *Bacillus subtilis* GB03 could trigger induced systemic resistance of *Arabidopsis* seedlings by activating endogenous ethylene synthesis and signalling against Erwinia carotovora. Apart from that, a study using Affymetrix Arabidopsis AG GeneChip revealed that the volatiles from bacterial strain GB03 exhibited upregulation of over 600 different transcripts, and these transcripts encode proteins with various functions, including cell wall modification, primary and secondary metabolism, stress response, and hormonal regulation (Zhang et al., 2007). Lately, there has been an increased understanding of the volatile compounds produced by bacteria, including long-chain hydrocarbons such as tridecane and hexadecane, C4 alcohols like 2,3-butanediol and ethyl propionate, and indole derivatives that have been found to be highly effective in inducing plant disease resistance (Farag et al., 2013; Lee et al., 2012). However, there are still large gaps in our knowledge concerning the mechanism of microbial VOCs in plant disease resistance, especially in postharvest fruits.

Tomato (*Solanum lycopersicum* L.) are not only horticultural crops of significant economic value worldwide but also classic model plants for studying the interactions between pathogens and host immune response (Abbasi et al., 2021). We previously found that VOCs emitted from *Burkholderia cenocepacia* ETR-B22 exhibited strong broad-spectrum antagonistic activity (Chen et al., 2020), considerably suppressed postharvest grey mould infection (Chen et al., 2022). Meanwhile, the bacterial VOCs emitted from ETR-B22were used as elicitors to induce resistance against *B. cinerea* in preliminary test, and the results were positive. Accordingly, the aims of this research were (a) to evaluate the effect of VOCs on the induction of disease resistance against grey mould in tomato fruit, (b) to investigate the influence of defence-related enzymes and the response gene expression of hormone signalling pathways, and (c) to explore the regulatory mechanism of VOCs-mediated post-harvest disease resistance in the transcriptome and metabolome. These results may further reveal the mechanism of induced disease resistance of tomato fruit by MVOCs and provide a novel theoretical basis for the safe and efficient control of postharvest diseases in tomato and other crops.

## 2 Materials and methods

### 2.1 Microorganisms and plant materials

VOCs were produced by *B. cenocepacia* ETR-B22 (hereafter referred to as ETR-B22), which was isolated from the roots of wild *Sophora tonkinensis* Gagnep. Based on our previous research, molecular identification of strain B22 has been conducted, and sequences of the 16S rRNA (accession numbers:MT712217) and *recA* gene (accession numbers: MT708133) were available in the NCBI repository (Chen et al., 2020). ETR-B22 was cultured on Tryptic Soy Agar (TSA) medium for 24 h at 28°C when required. Individual bacterial colonies were grown in Tryptic Soy Broth (TSB) medium in a shaking incubator (180 rpm) overnight, and fresh bacteria were collected by centrifugation and resuspended in phosphate buffer solution (PBS) to adjust the final concentration to 10^7^ colony forming units (CFU) mL^-1^. The pathogen *B. cinerea* was purchased from the Guangdong Microbial Culture Collection Centre (preservation number: GDMCC 3.47). The fungi were cultured on potato dextrose agar (PDA) medium at 28°C for 15 d. Thereafter, the spores were harvested in PBS. Spore concentration was measured using a haemocytometer and adjusted to 10^5^ CFU mL^-1^.

Tomato fruits (*Solanum lycopersicum* L.) were harvested from the greenhouse of Guangxi University, Nanning, China, and immediately taken to the laboratory. Healthy fruits of similar size at the red stage were selected, surface-sterilised in a 0.1% sodium hypochlorite solution for 2 min, and rinsed thoroughly with water.

### 2.2 Fruit treatment and sampling

**Figure 1:**
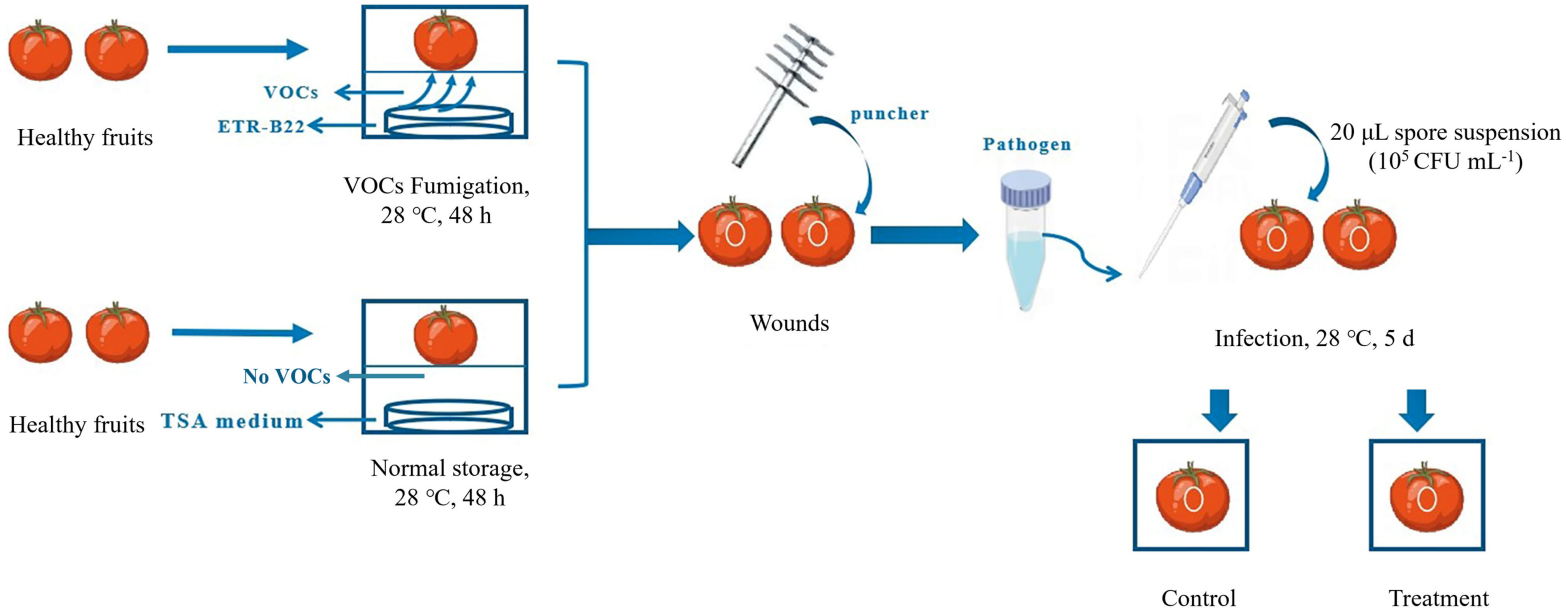
Diagrammatic illustration of the stages in microbial volatile organic compounds (VOCs) treatment. In the illustration, “Control” represented that the toamto fruits inoculated with *B*. *cinerea* without VOCs fumigation, while “Treatment” represented that toamto fruits fumigated with VOCs and inoculated with *B*. *cinerea*.

The equipment used for the induced resistance test in this study was a sealed square plastic box (8 × 8 × 8 cm) with a perforated separator. For the VOCs resistance-inducing treatment (hereinafter referred to as Treatment), the TSA medium was poured into the bottom of a sterilised plastic box, while a 20 μL suspension of ETR-B22 was sprayed on the TSA medium and incubated at 28 °C for 12 h as resistance-inducing sources of VOCs. After incubation, tomatoes were quickly placed on the separator of the box and kept non-contact with the medium. Subsequently, the plastic box was sealed with solid tape to prevent leaks and fumigated for 48 h at 28°C under dark conditions. For the test control (hereinafter referred to as Control), sterile water was sprayed onto the TSA medium, which meant no fumigation with VOCs on tomato. Afterward, the wound (4 mm × 2 mm) was made with a sterile borer on the equatorial region of each fruit, inoculated with 20 μL of *B. cinerea* spore suspension (10^5^ CFU mL^-1^), and stored in a new plastic box at 28°C. A representation of the process of resistance induction in tomatoes is shown in **Figure 1**. Each treatment was performed in triplicate, with 60 fruits per treatment. Flesh samples of the “Treatment” and “Control” fruit from 3 to 5 mm under epidermis around the equator were collected at 0, 1, 2, 3, 4, and 5 d. The samples were rapidly frozen in liquid nitrogen, packed in aluminium foil, and stored at −80°C for the following experiment.

### 2.3 Disease incidence and lesion diameters

Sixty fruits were randomly divided into five groups of 12 fruits each. The disease incidence and lesion diameter of 12 fixed fruits were measured at 1, 2, 3, 4, and 5 d intervals after inoculation. The disease incidence was calculated as the number of infected fruit/total number of fruits × 100%, and lesion size of each infected fruit was measured using vernier caliper.

### 2.4 Measurement of defence-related enzyme activities

Peroxidase (POD), phenylalanine ammonia-lyase (PAL), and polyphenol oxidase (PPO) levels were determined as described by Duan et al. (2024). The PAL, POD, and PPO activities were expressed as U g^−1^ FW, where U= 0.01 OD_290_ h^−1^, U = 0.01 OD_470_ min^−1^, and U = 0.01 OD_420_ min^−1^, respectively. Ascorbic acid peroxidase (APX) activity was measured as described by Tang et al. (2020). One unit of APX activity was defined as an increase in the absorbance of 0.01 at 290 nm per min. Chitinase (CHI) and β-1,3-glucanase (GLU) activity was assessed according to the method of Sun et al. (2024): one unit of CHI activity required to produce 1 μmol N-acetylglucosamine of reducing chitin per gram FW per h, and one unit of GLU activity was defined as the amount of enzyme needed to break down fucoidan per s to produce 1 × 10^-9^ mol of glucose.

### 2.5 Real-time quantitative polymerase chain reaction analysis of hormone biosynthesis and signalling pathways

Total RNA was extracted from frozen tissues from two individual experiments (*Section 2.2*) using a TRIzol reagent kit (TransGen Biotech, Beijing City, China). RNA quality and integrity were assayed using spectrophotometry (Thermo Fisher Scientific, Waltham, MA, USA) and agarose gel electrophoresis, respectively. Total RNA (1 μg) was used for cDNA synthesis using a TansScript All-in-one First-Strand cDNA Synthesis Kit (TransGen Biotech). Subsequently, diluted cDNAs were used for RT-qPCR by a LightCycler96 system (Roche, Basel, Switzerland), following the instructions of the PerfectStart^®^ Uni RT&qPCR Kit (TransGen Biotech). The sequence of target genes in the enzyme synthase and hormone signalling pathways were obtained from the specific sequence of *S. lycopersicum* deposited in NCBI GenBank, and the gene-specific primers were designed with Primer Premier 6.0 software (PREMIER Biosoft International, Palo Alto, CA, USA) and list in (**Supplementary Table 1**). Gene expression with three biological replicates was calculated according to the comparative 2^−△△CT^ method and was normalised to the corresponding reference genes levels.

### 2.6 Transcriptomic and metabolomics analysis

#### 2.6.1 Sample preparation

Combining disease development in fruits and the changes in enzyme activities at different time points, the third day after inoculation was considered the key stage. Individual experiments were conducted to resolve the mechanisms of induced resistance based on transcriptomic and metabolomic analyses. Three groups of experiments were designed as follows: (a) a control group (CK), where the healthy tomato fruit without any treatment (VOCs fumigation and pathogen inoculation were not performed); (b) inoculated fruit after VOCs induction treatment (BF), where the resistance of fruits was induced by VOCs according to the method described in *Section 2.2* and then inoculated with 20 μL of *B. cinerea* spore suspension (10^5^ CFU mL-1); and (c) inoculated fruit without VOC induction treatment (F0) where the fruit was inoculated with 20 μL of *B. cinerea* spore suspension with the same concentration but was not induced with VOCs. Seventy-two hours after inoculation, a total of 30 fruits from three replicates of each treatment were sampled, and 6 g of fruit flesh per replicate was prepared and stored at -80°C for metabolite determination and RNA extraction.

#### 2.6.2 Transcriptome sequencing, assembly, and analysis

RNA extraction, library construction, and sequencing were performed by Shanghai Majorbio Bio-Pharm Technology Co., Ltd. (Shanghai, China). RNA-seq data were analysed according to the method described by Zhang et al. (2019). In brief, to ensure data quality, the fastx_toolkit (Version 0.0.14) was utilised to clean the raw sequence reads. Specifically, low-quality bases at the 3’ end were removed, along with any ambiguous N bases. Additionally, all low-quality read lengths, including short reads (<20 nt), empty reads, and ncRNA reads, were eliminated.The clean reads were aligned with the *S. lycopersicum* v4.0 reference genome (ftp://ftp.solgenomics.net/genomes/Solanum_lycopersicum/Heinz1706/assembly/build_4.00/) using HISAT2 software (Version 2.1.0). Gene Ontology (GO), Kyoto Encyclopedia of Genes and Genomes (KEGG), and Non-Redundant Protein (Nr) databases were utilized for the functional identification and annotation of genes. Gene expression levels were calculated as transcripts per kilobase million (TPM). The R package DESeq was used to identify differentially expressed genes (DEGs) with the threshold of the adjusted P value < 0.05 and |Log_2_ Fold Chang| ≥ 2. To infer the supposed function of the DEGs, KEGG pathway enrichment analysis was conducted using the ClusterProfiler R package. The methods of qRT-PCR validation of selected DEGs were the same as in section 2.5. The gene-specific primers were listed in **Supplementary Table 4.**

#### 2.6.3 Widely targeted metabolomics profiling

Sample pretreatment, extraction, and widely targeted metabolomic assays based on the UPLC-QQQ-MS (UPLC, SHIMADZU Nexera X2; MS, Applied Biosystems 4500 QTRAP) system were performed by Wuhan Metware Biotechnology Co., Ltd. (Wuhan, China). For qualitative analysis, primary and secondary mass spectrometry data were obtained by searching the Metware in-house database. Briefly, the column employed in this study was an Agilent SB-C18, with dimensions of 1.8 µm and 2.1 mm × 100 mm. The mobile phase composition consisted of solvent A (pure water with 0.1% formic acid) and solvent B (acetonitrile with 0.1% formic acid). Using a gradient program, the sample was measured with an initial condition of 95% A and 5% B. Within 9 minutes, the proportion of B phase linearly increased to 95% and was maintained at 95% for 1 minute. Then, within 2 minutes, the proportion of the B phase decreased to 5% and remained at 5% until 14 minutes. During the operation, the column temperature was set at 40°C, and the injection volume was 4 μL. Mass spectrometry conditions were as follows: the electrospray ionisation source temperature was set to 550°C; the ion spray voltage was set to 5500 V (positive ion mode)/-4500 V (negative ion mode); gas I, gas II, and curtain gas pressures were set to 50, 60, and 25 psi, respectively; collision Gas (CAD) was set to “high”. Instrument tuning and mass calibration are performed using 10 and 100 μmol/L polyethylene glycol solutions in QQQ and LIT modes, respectively. QQQ scans are conducted in multiple reaction monitoring (MRM) mode, with the collision gas (nitrogen) set to medium. The declustering voltage (DP) and collision voltage (CE) for each MRM ion pair are optimised through further adjustment. A specific set of MRM ion pairs is monitored for each eluting metabolite in each period. Quantitative analysis of the identified metabolites was performed in the MRM mode. Data were subjected to multivariate statistical analysis using R software (www.r-project.org/), and principal component analysis (PCA) and orthogonal partial least square-discriminant analysis (OPLS-DA) were employed. The differential accumulated metabolites (DAMs) were selected based on variable importance in the projection (VIP) values > 1, |log2(FC)| ≥ 1 or ≤ 0.5, and P < 0.05. The metabolic pathways of selected DAMs were identified using the KEGG database.

### 2.7 Statistical analysis

Statistical analyses were performed using the IBM SPSS Statistics version 22 (SPSS Inc., Chicago, USA). All experimental data were expressed as mean and standard deviation values, and differences at *P* < 0.05 were considered significant when compared using a Student’s t-test.

## 3 Results

### 3.1 VOCs fumigation induced disease resistance of postharvest tomato fruit against grey mould

**Figure 2:**
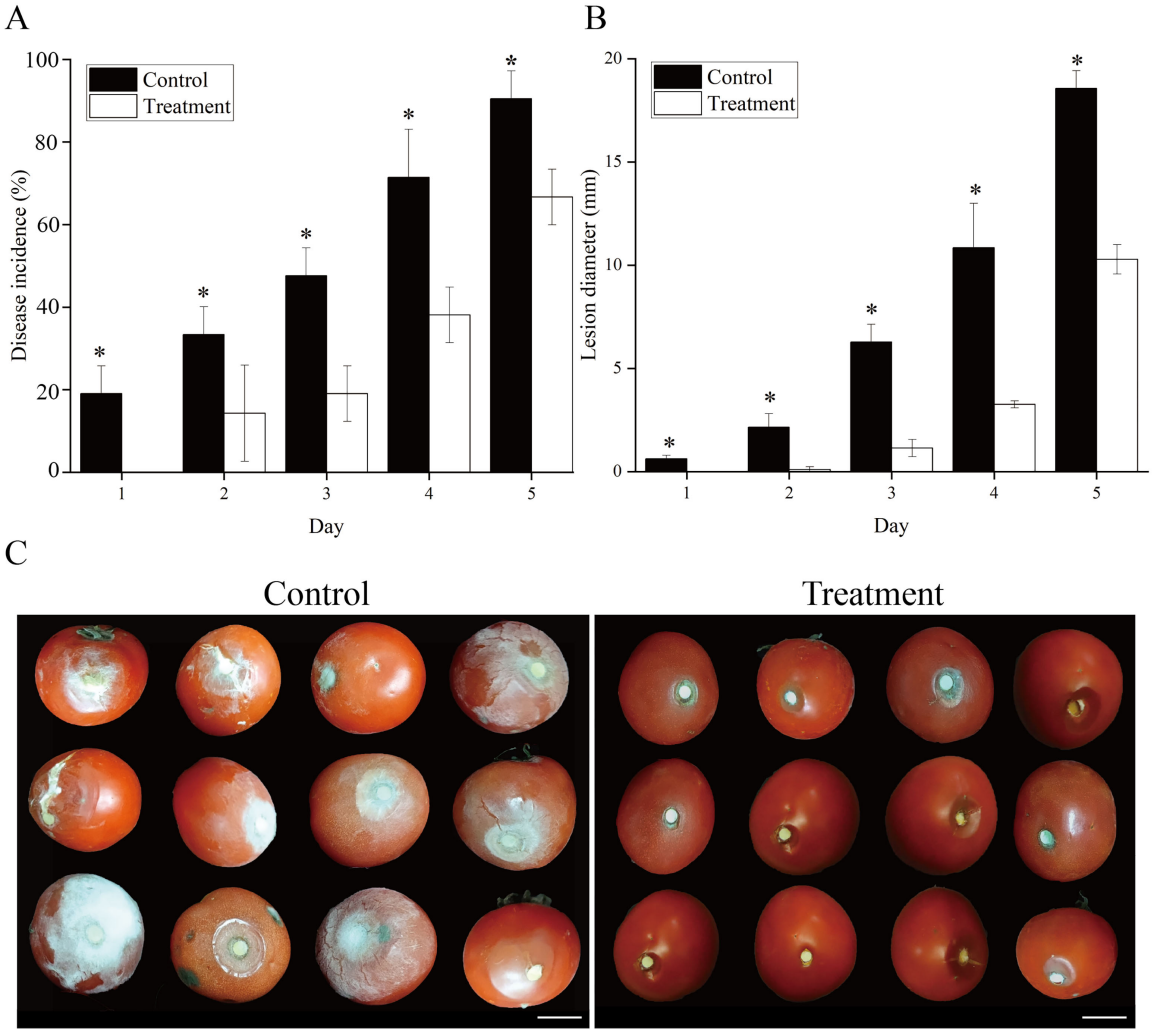
Effect of VOCs treatment on induced resistance of tomato fruit inoculated with *Botrytis cinerea*. The tomato fruits were fumigated with VOCs in a seal plastic case for 48 h and inoculated with 20 μL of *B. cinerea* at 1×10^5^ spores mL^−1^. Disease incidence rate **(A)** and lesion diameter **(B)** of infected fruits were investigated during storage on Day 5. The data are presented as the means of three replications, and error bars indicate the standard error of the mean values. Asterisk in the figure indicated that significant differences between two treatment groups at the same time point according to the Student’s t test (*P *< 0.05). Control: fruits inoculated with *B. cinerea* without VOCs fumigation. Treatment: fruits fumigated with VOCs and inoculated with *B. cinerea*.

The tomato fruits treated with VOCs showed effective resistance to *B. cinerea* (**Figure 2**). Disease incidence and lesion diameter in tomato fruits inoculated with *B. cinerea* were decreased throughout the storage period after VOCs induction. At each time interval, the disease incidence rate and lesion diameter differed significantly (P < 0.05) between the VOC-treated and control groups. When storage ended, VOCs treatment reduced disease incidence and lesion diameter to 25.92% and 54.37%, respectively, compared to the control (**Figures 2A, B**).

### 3.2 VOCs activated different functions of defence-related enzyme activity

**Figure 3:**
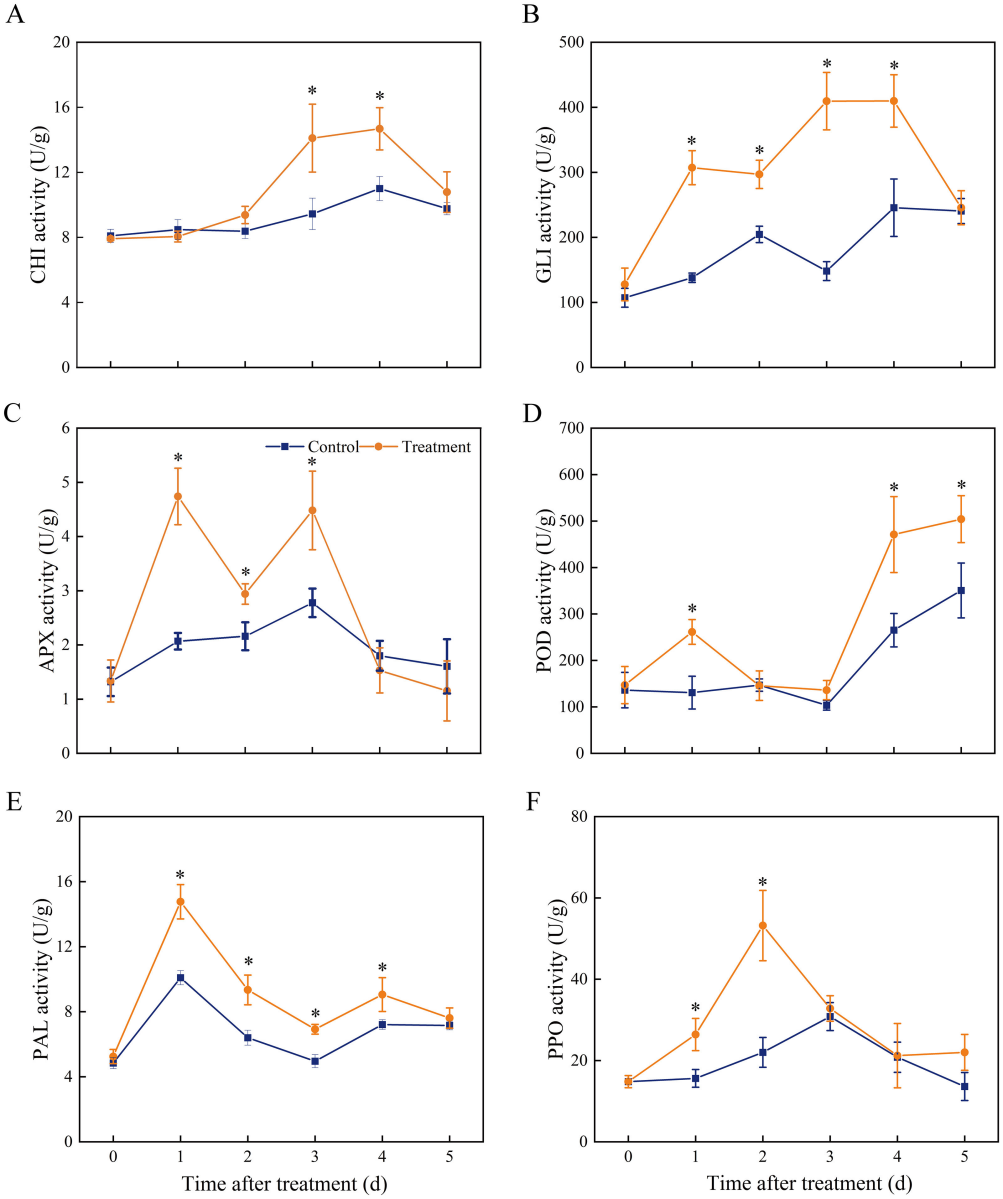
Effect of VOCs on the activities of **(A)** chitinase, **(B)** β-1,3-glucanase, **(C)** ascorbate peroxidase, **(D)** peroxidase, **(E)** phenylalanine ammonia-lyase, and **(F)** polyphenol oxidase in tomato fruits. Values are the mean ± standard deviation (SD) of three replicates. Asterisk in the figure indicated significant differences between two treatment groups at the same time point according to the Student’s t test (*P* < 0.05).

**Figure 4:**
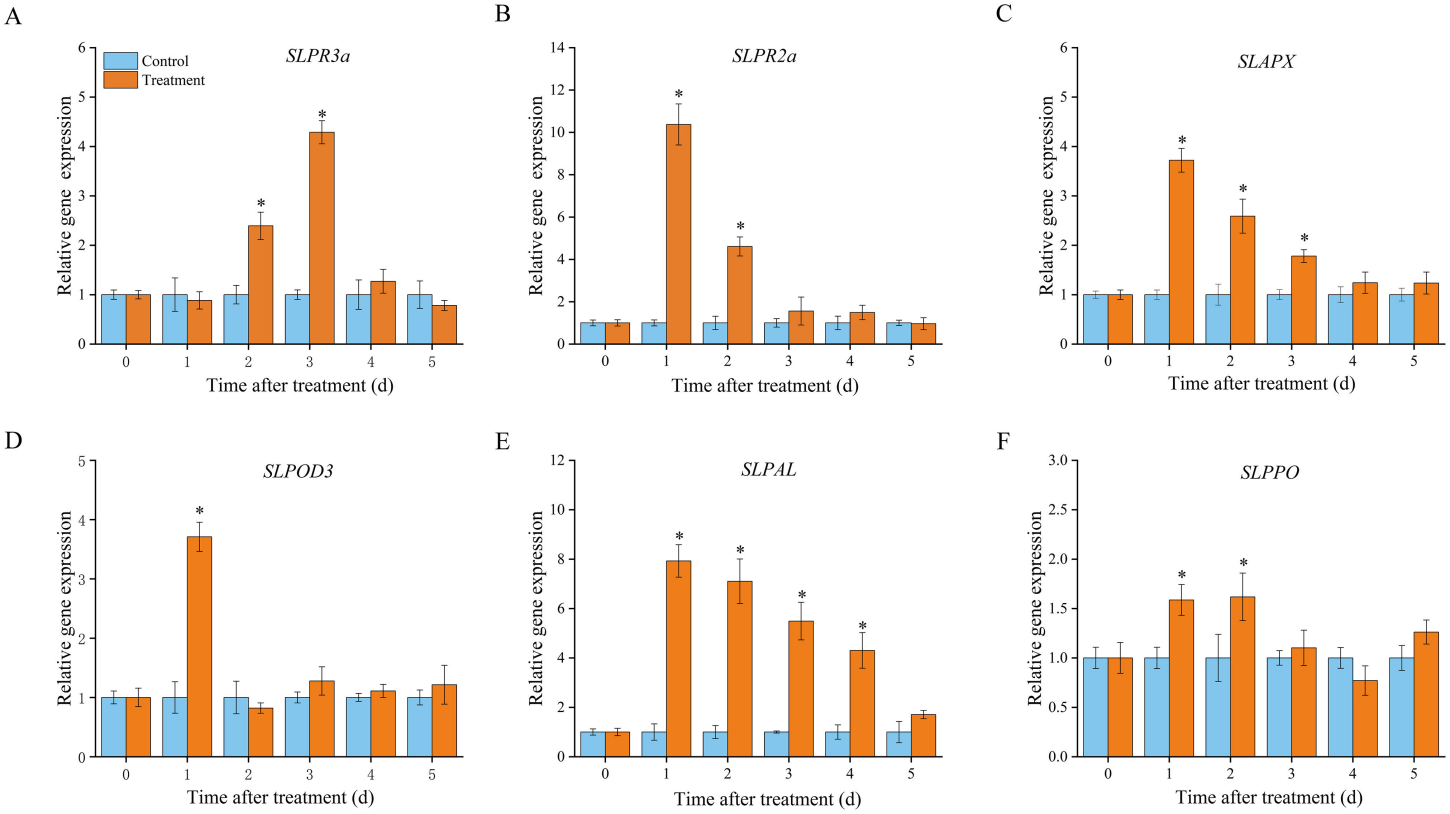
Quantitative RT-PCR analysis for the encoding genes expression of chitinase (*SlPR3a*; **(A)**,β-1,3-glucanase (*SlPR2a*; **(B)**, ascorbate peroxidase (*SlAPX*; **(C)**, peroxidase (*SlPOD3*; **(D)**, phenylalanine ammonia-lyase (*SlPAL*; **(E)**, and polyphenol oxidase (*SlPPO*; **(F)** in fruit tissue. Values are the mean ± standard deviation (SD) of three replicates. Asterisk in the figure indicated significant differences between two treatment groups at the same time point according to the Student’s t test (*P* < 0.05). Control: fruits inoculated with *B*. *cinerea* without VOCs fumigation. Treatment: fruits fumigated with VOCs and inoculated with *B*. *cinerea*.

We examined the activities of defence enzymes with different functions, including disease resistance-associated proteins (GLU and CHI), antioxidant enzymes (APX and POD), and phenylpropane pathway enzymes (PAL and PPO) (**Figure 3**). Compared to that in the control samples, CHI activity in the treatment samples had no effect within 2 d of inoculation but considerably increased at 3–4 d compared to the control (**Figure 3A**). Increased expression of *SLCHI* was also observed in treated fruits at 2 and 3 d, with a 1.49- and 1.31- fold increase compared to that in the control, respectively (**Figure 4A**). Marked increases in GLU activity were observed in the medium after 4 days of storage in treated samples (**Figure 3B**), and the expression level of *SLGLU* in treated fruits increased by 10.37-fold on Day 1 and 1.58-fold on Day 3 of storage (**Figure 4B**). APX activity showed double peaks in VOCs-treated fruits, one on Day 1 and the other on Day 3 (**Figure 3C**). There was an obvious accumulation of *SLAPX* from Day 1 to Day 3, and the levels were 3.72-, 2.59-, and 1.78-fold greater on Days 1, 2, and 3, respectively, than those in the control group (**Figure 4C**). Higher POD activity was recorded in treated fruits at Days 1, 4, and 5 (**Figure 3D**), but the expression of *SLPOD* was only substantially upregulated at Day 1 (**Figure 4D**). PAL activity in VOCs-treated fruits rapidly reached a peak value on Day 1 and then decreased gradually until Day 5 but remained higher than that of the control on Days 2–4 (**Figure 3E**). Similarly, the expression of PAL in fruit treated with VOCs showed the same variation trend and likewise peaked on Day 1 with a 7.92-fold higher expression than that in the control (**Figure 4E**). PPO activity increased in the VOCs-treated fruits, peaked on Day 2, and then declined (**Figure 3F**). The values at Days 1 and 2 were 1.69- and 2.41-fold higher than those in the control groups, respectively. Further RT-qPCR also showed that *SLPPO* expression in the VOCs treatment was significantly higher (P < 0.05) than that in the control on storage Days 1 and 2, and no significant difference was observed from storage Days 3–5 (**Figure 4F**).

### 3.3 VOCs exposure promoted SA and JA biosynthesis and signalling but suppressed ET signalling

**Figure 5:**
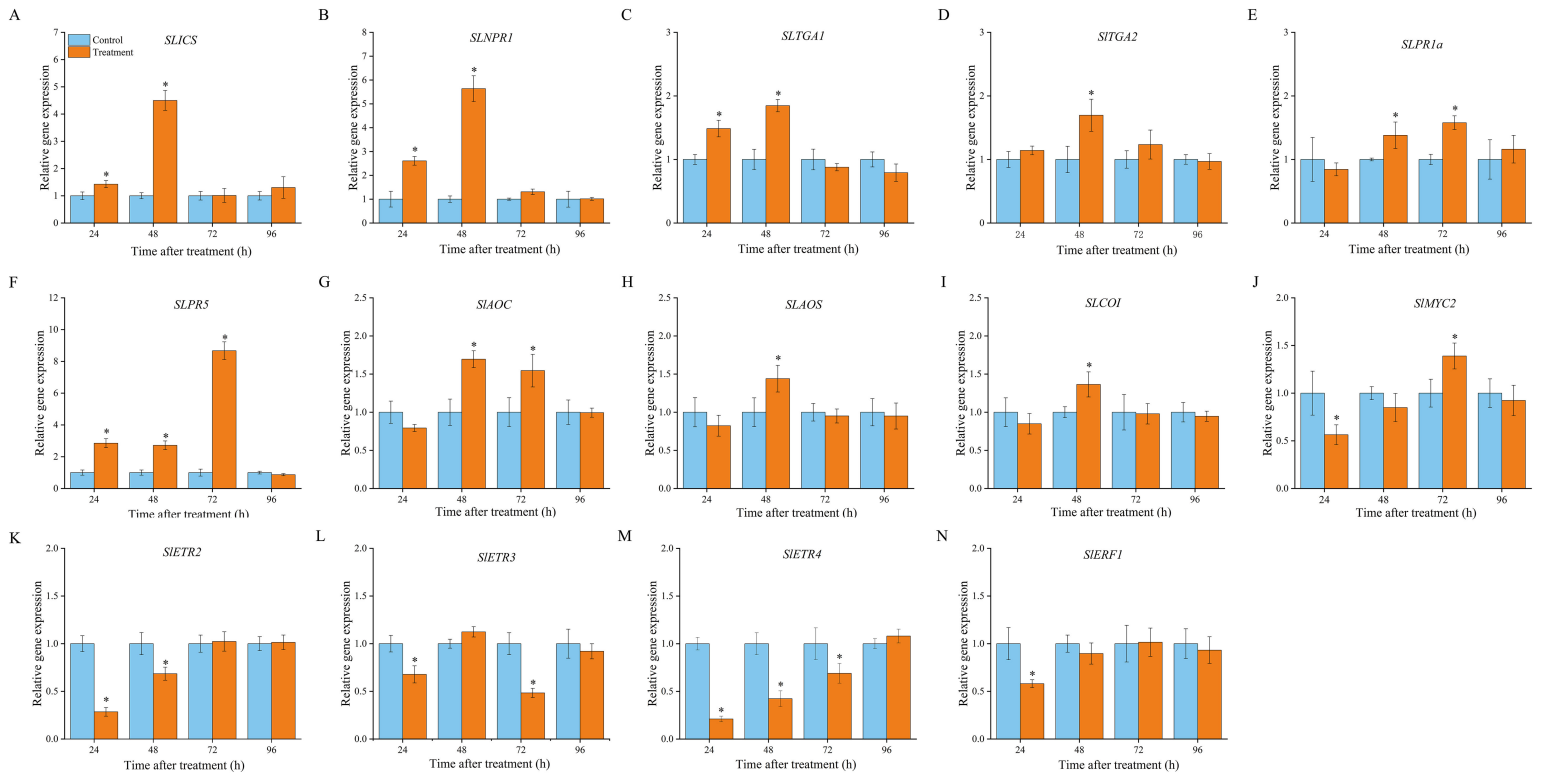
Involvement of phytohormone-mediated signaling pathway in VOCs-induced resistance of tomato. The gene expression in SA signaling pathway of isochorismate synthase (*SlICS*; **(A)**, the non-inducible pathogenesis-related 1 (*SlNPR1*; **(B)**, TGA transcription factors 1a (*SlTGA1a*; **(C)**, TGA transcription factors 2 (*SlTGA2*; **(D)**, pathogenesis-related protein gene 1b1 (*SlPR1b1*; **(E)**, and pathogenesis-related protein gene 5 (*SlPR5*; **(F)**; the gene expression in JA signaling pathway of allene oxide cyclase (*SlAOC*; **(G)**, allene oxide synthase (*SlAOS*; **(H)**, COR-insensitive 1(*SlCOI*; **(I)**, basic helix-loop-helix MYC2 (*SlMYC2*; **(J)**; the gene expression in ET signaling pathway of ethylene receptor 2 (*SlETR2*; **(K)**, ethylene receptor 3 (*SlETR3*; **(L)**, ethylene receptor 4 (*SlETR4*; **(M)**, and ethylene responsive factor 1 (*SlERF1*; **(N)**. Values are the mean ± standard deviation (SD) of three replicates. Asterisk in the figure indicated significant differences between two treatment groups at the same time point according to the Student’s t test (*P* < 0.05).

Isochorismate synthase (ICS) and PAL are the two key enzymes responsible for SA biosynthesis. As described in *Section 3.2*, *SLPAL* expression was markedly increased in VOCs-treated fruits compared with that in the control group (**Figure 3E**). Meanwhile, VOCs treatment induced an increase in *SlICS* expression in the inoculated fruit, with 1.42- and 4.51-fold upregulation compared to the control group (**Figure 5A**). In the SA signalling pathway, non-inducible pathogenesis-related 1 (NPR1) is considered a master regulator of SA signalling and interacts with TGA transcription factors, ultimately leading to the activation of SA-dependent responses (Ding et al., 2022). **Figure 4A** shows that the expression levels of *SLNPR1* increased in the VOCs-treated samples, showing a significant increase from 24 h to 72 h (P < 0.05), to levels 2.61-, 5.64-, and 1.31-fold higher than those in the control samples (**Figure 5B**). The expression level of *SlTGA1* in the VOCs-treated samples increased at 24 h and 48 d (**Figure 5C**), and *SlTGA2* was highly expressed in the VOCs-treated samples at 48 h relative to that in the control samples (**Figure 5D**). VOCs treatment enhanced the expression of two PR genes (*SlPR1* and *SlPR5*) (**Figures 5E, F**). A spike in the expression level of *SlPR5* in VOCs-treated samples was recorded at 72 h, which was 8.67 times higher than that in the control samples. These results indicate that VOCs exposure in fruits activates SA biosynthesis and signalling to enhance fruit resistance.

In the JA pathway, allene oxide cyclase (AOC) and allene oxide synthase (AOS) are the functional enzymes involved in JA biosynthesis and are mainly responsible for the cyclisation of fatty acid hydroperoxide (Carvalhais et al., 2017). Plants perceive external pathogens to generate JA-Ile, which promotes interactions between COI1 and JAZ proteins. Subsequently, JAZ proteins are degraded after being transferred to the 26S proteasome, and transcriptional factors, such as MYC, are released simultaneously to activate downstream gene expression (McSteen and Zhao, 2008). As shown in **Figure 4B**, VOCs treatment enhanced the expression of *SlAOC* at 48 and 72 h, but the expression level was remarkably downregulated 24 h after inoculation in comparison with that in the control samples (**Figure 5G**). *SlAOS* and *SlCOI* were highly expressed at 48 h in the VOCs-treated samples, with the abundance of gene transcription 1.44- and 1.34-fold greater than that of the control, respectively (**Figures 5H, I**). VOCs treatment resulted in the clear suppression of *SlMYC2* expression at 24 h but triggered an evident elevation in the accumulation of SlMYC2 at 72 h to a level 1.38-fold greater than that in the control samples (**Figure 5J**).

Four genes involved in the ET signalling pathway were markedly downregulated in the VOCs-treated fruits at different time points during storage. In the first 72 h, the expression levels of *SLETR4* were always lower in the VOCs-treated samples than in the corresponding control samples (**Figure 5M**). A minimum expression, 0.21-fold lower than that in the control samples, was recorded at 24 h. Expression of *SLETR2* in VOCs-treated fruits followed a pattern similar to that of *SLETR4* (**Figure 5K**). A value 0.29-fold lower than that of the control samples was observed at 24 h. The expression levels of *SLETR3* decreased in the VOCs-treated samples at 24 and 72 h, with a 0.64- and 0.84-folds decrease compared to those in the control samples, respectively (**Figure 5L**). *SLERF1* was expressed at low levels in VOCs-treated fruits compared to that the control group at 24 d. Beyond 48 h, its expression level was approximately the same as that in the control group (**Figure 5N**).

### 3.4 Metabolomic analysis proved the accumulation of phenolic compounds relating to VOCs-induced tomato fruit resistance

**Figure 6:**
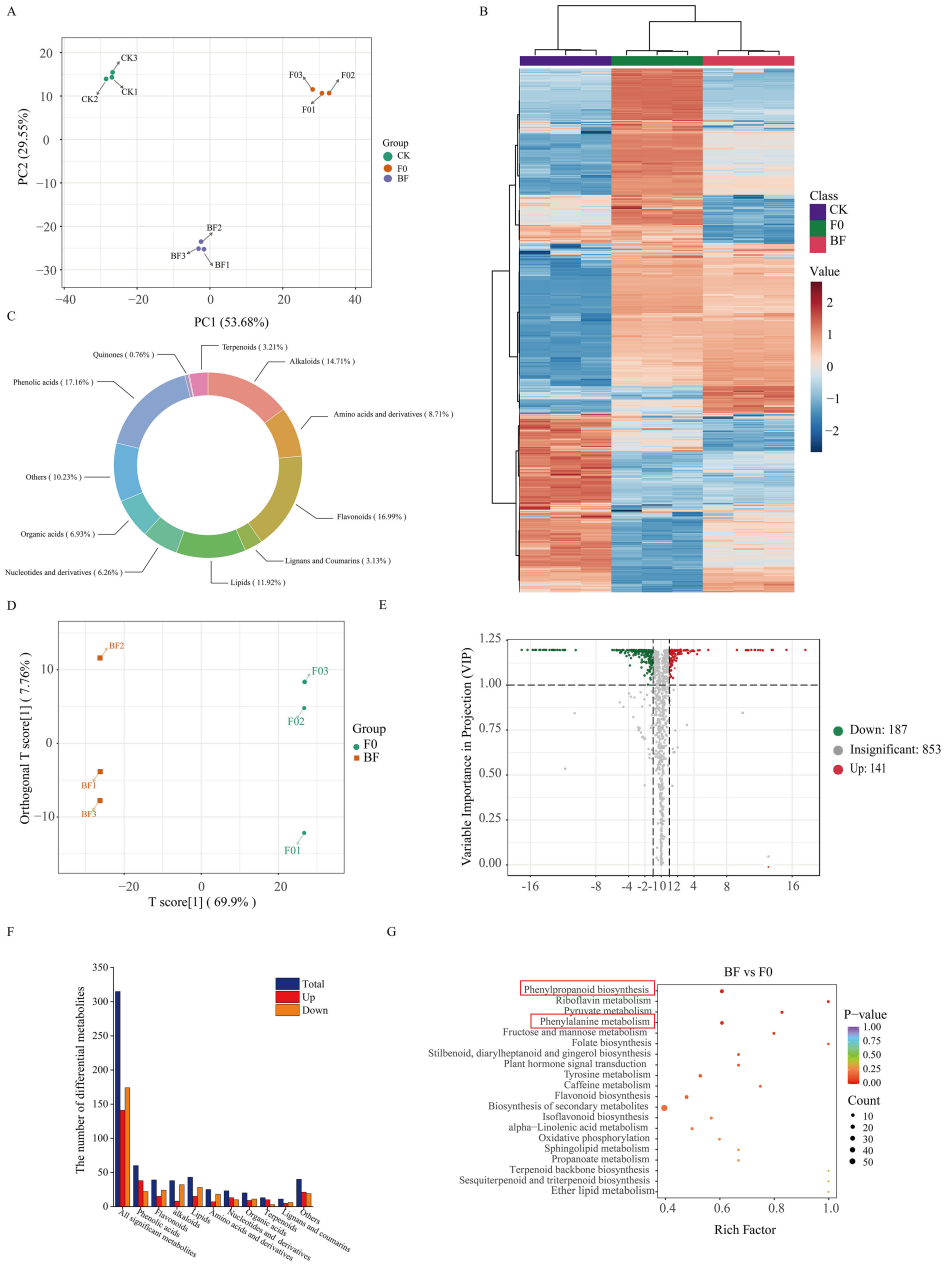
Widely targeted metabolomics analysis in tomato fruits treated with volatile organic compounds. **(A)** Principal component analysis (PCA) of metabolites identified in tomato fruit samples from three treatments. **(B)** Unsupervised hierarchical cluster analysis of all metabolites identified in samples from the three treatments. The heatmap colours indicate the relative content of each metabolite, from low (blue) to high (red). **(C)** Pie chart depicting the biochemical classification of the metabolites identified among three treatments. **(D)** Orthogonal projections to latent structures discriminant analysis (OPLS-DA) score plots of BF VS F0 group. **(E)** Volcano plot of differential metabolites in BF vs. F0 group. **(F)** Classification and the number of the identified differentially accumulated metabolites in BF vs. F0. **(G)** KEGG enrichment analysis of differential accumulated metabolites in BF vs. F0. CK: healthy fruits not fumigated with VOCs without *B*. *cinerea* inoculation; BF: Inoculated fruit with VOCs induction; F0: Inoculated fruit without VOCs induction.

**Figure 7:**
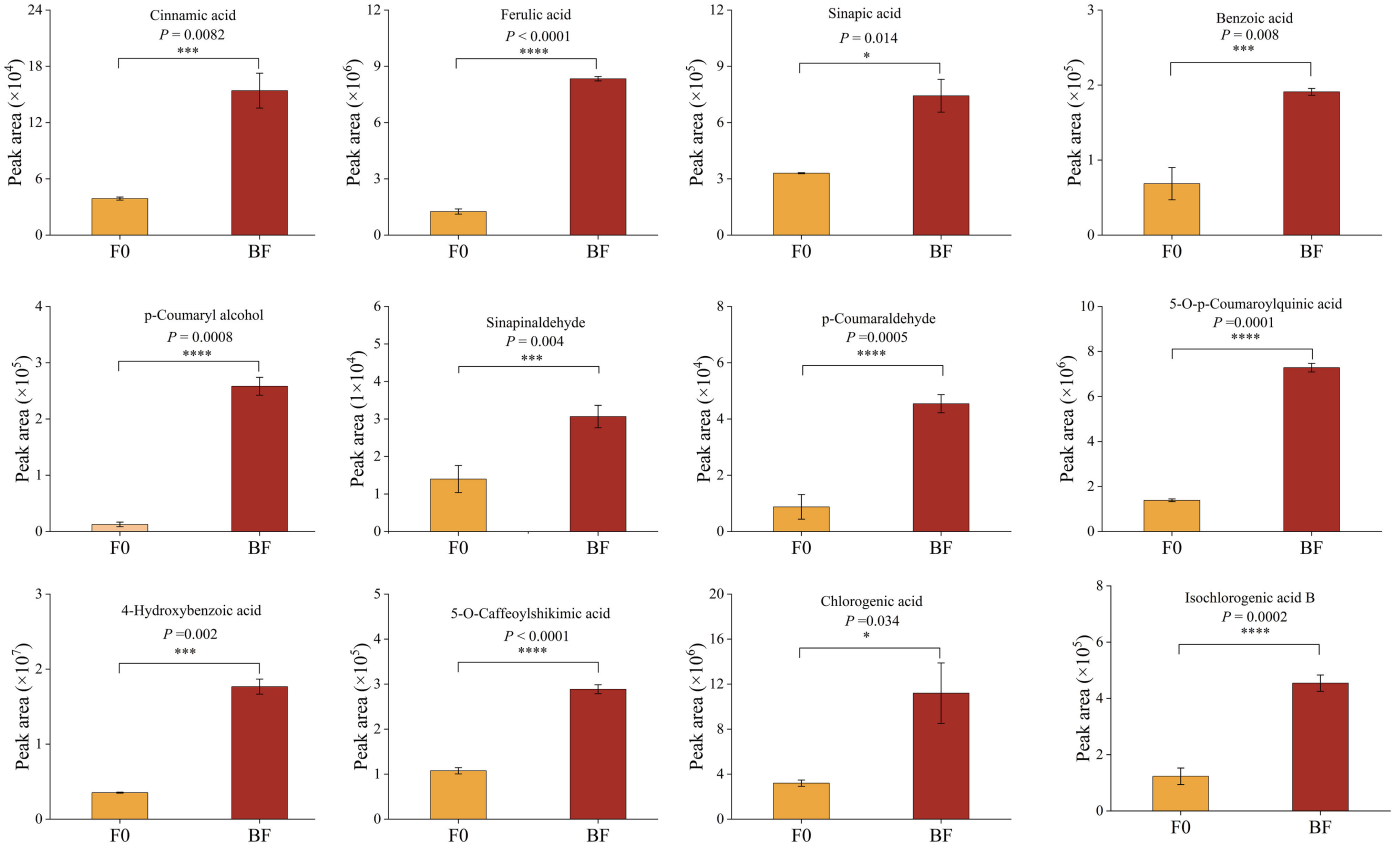
Key differentially accumulated metabolites in tomato fruits treated with volatile organic compounds. Asterisk in the figure indicated significant difference between BF and F0 group. *Significant difference at *P* <  0.05; **Significant difference at *P* <  0.01; *** Significant difference at *P *<  0.001; **** Significant difference at P <  0.0001 based on Student’s t test. BF: Inoculated fruit with VOCs induction; F0: Inoculated fruit without VOCs induction.

To gain a comprehensive understanding of resistance-related metabolic changes, samples from the CK, BF, and F0 groups after inoculation with pathogenic fungi were used for widely targeted metabolomic sequencing (**Figure 6**). PCA and hierarchical clustering revealed considerable separation among the different treatments (**Figures 6A, B**). A total of 1,181 metabolites were detected and divided into 11 groups (**Figure 6C**). OPLS-DA was employed to maximise the class discrimination between F0 and BF tomatoes (**Figure 6D**). R^2^Y (close to 1) and Q^2^ (> 0.5) with high values indicated the goodness of fit and predictive capability of the models, and this model generated VIP scores (**Supplementary Figure 1**). A total of 328 metabolites were identified as being significantly different between BF and F0, including 187 downregulated and 141 upregulated metabolites (**Figure 6E**). KEGG enrichment analysis showed that DAMs were mainly enriched in “phenylpropanoid biosynthesis” and “phenylalanine metabolism” (**Figure 6G**). The amount of phenolic acids with higher accumulation in the BF treatment increased considerably (**Figure 6F**; **Supplementary Table 2**), indicating that phenolic acid was the main contributor to the improvement in tomato resistance in this experiment. Among the markedly accumulated phenolic acids in the VOCs-treated samples, the abundance of substrates of the phenolic acid metabolism pathway, including cinnamic acid, sinapic acid, ferulic acid, p-coumaryl alcohol, sinapinaldehyde, and p-coumaraldehyde, in BF was higher than that in F0 (**Figure 7**). Meanwhile, the contents of some compounds with antioxidant or antifungal activity, including two major groups of metabolites, hydroxybenzoic acid and hydroxycinnamic acid, such as chlorogenic acid, isochlorogenic acid A, 4-hydroxybenzoic acid, 2,5-dihydroxybenzoic acid, 2,5-dihydroxybenzaldehyde, 5-O-p-coumaroylquinic acid, 5-O-caffeoylshikimic acid, 2-hydroxycinnamic acid, and α-hydroxycinnamic acid, also rapidly increased after VOCs treatment (**Figure 7**; **Supplementary Table 2**). Therefore, we inferred that they were potential contributors to disease resistance.

### 3.5 Transcriptomic analysis revealed phenylalanine biosynthesis and metabolism pathways mediating VOCs-induced tomato fruit resistance

**Figure 8:**
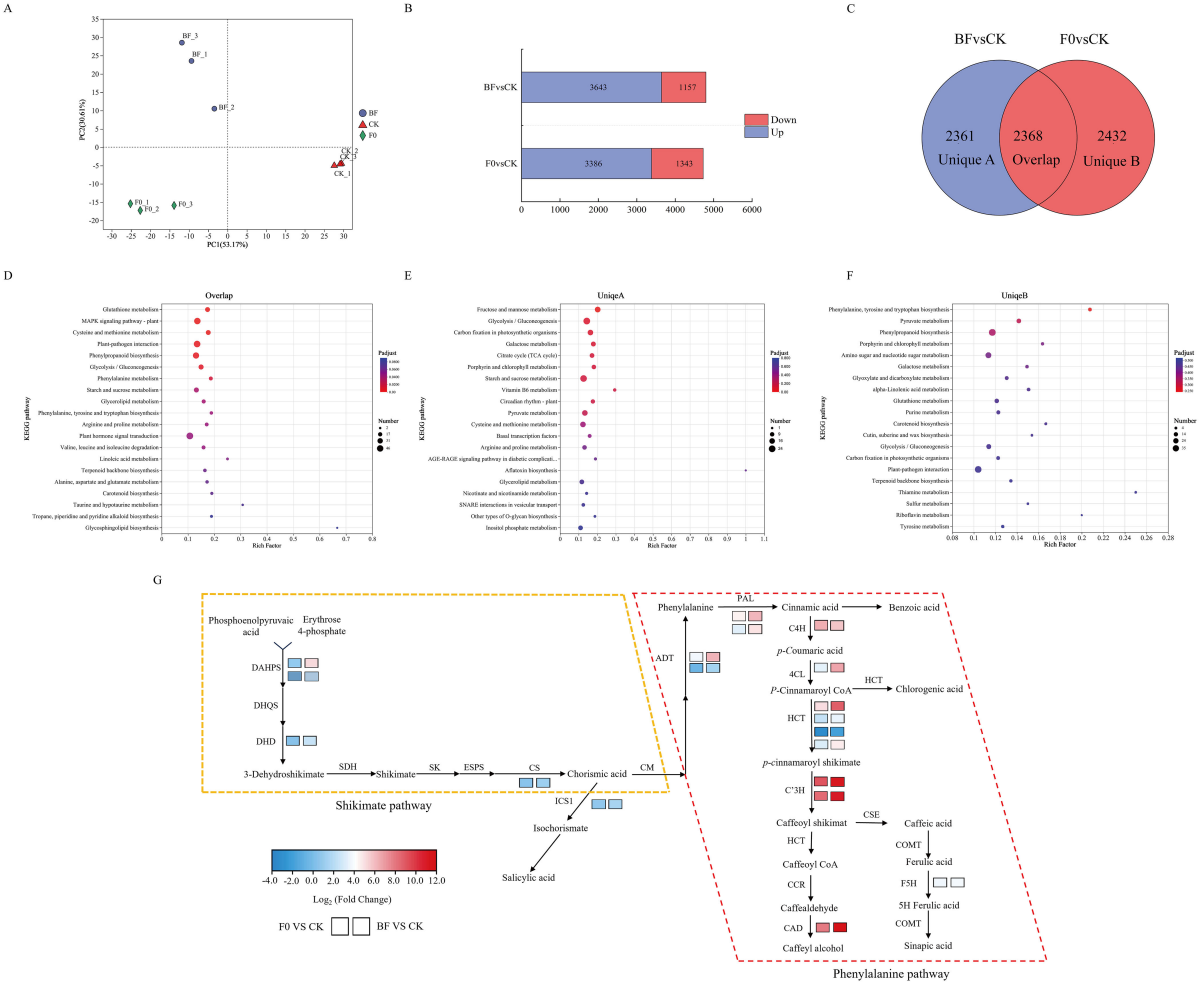
Transcriptional analysis of tomato fruits responsive to the control (CK), Inoculated fruit without volatile organic compounds induction (F0), and inoculated fruit after microbial volatile organic compounds induction (BF) treatments. **(A)** PCA analysis of gene expression among sample groups. **(B)** The differentially expressed genes between the BF vs. CK and F0 vs. CK comparison groups. **(C)** Venn diagram of differentially expressed genes between the BF vs. CK and F0 vs. CK comparison groups. **(D)** KEGG pathway enrichment of overlapped DEGs between the BF vs. CK and F0 vs. CK comparison groups. **(E)** KEGG pathway enrichment of unique DEGs in BF vs. CK. **(F)** KEGG pathway enrichment of unique DEGs in F0 vs. CK. **(G)** The related genes involved in phenylalanine upstream metabolism and phenolic acids biosynthesis in BF and F0 groups. The box on the left represents the F0 vs. CK comparison group, and the box on the right represents the BF vs. CK comparison group. A log_2_ (Fold Change) > 1 indicates that the genes are upregulated, and a log_2_ (Fold Change) < −1 indicates that the genes are downregulated.

To understand the molecular mechanisms underlying the resistance of tomato fruits to VOCs, high-quality total RNA of the fruits stored for 72 h was prepared from the VOCs-treated fruits and control. cDNA libraries were constructed, amplified, and sequenced using Illumina sequencing. PCA of gene expression among different groups showed that the samples from different treatment groups were scattered, whereas the samples in each group were clustered, indicating that the responses of genes in each treatment group were considerably different and that the samples had good repeatability (**Figure 8A**). DEGs were identified based on the specific threshold (|log_2_ (Fold change) | ≥ 1 and FDR < 0.01). A total of 3,368 upregulated and 1,343 downregulated genes were identified in the F0 and CK groups. Moreover, 3,643 and 1,157 genes were upregulated and downregulated, respectively, in comparison to BF and CK (**Figure 8B**). According to the Venn diagram, 2,368 DEGs were overlapped in the comparisons between F0 and CK as well as between BF and CK (**Figure 8C**). KEGG enrichment analysis showed that the overlapping DEGs were all highly enriched in well-known disease resistance pathways, such as plant hormone signal transduction, plant–pathogen interaction, and MAPK signalling pathways (**Figure 8D**). The overlapping DEGs were also enriched in phenylpropanoid biosynthesis and phenylalanine metabolism, which can produce fungistatic substances. Compared with the CK samples, the unique DEGs of the BF and F0 samples after pathogen inoculation were enriched in different pathways. Among them, the unique DEGs of “F0” were enriched in the pathways unrelated to disease resistance (**Figure 8F**), while the unique DEGs of “BF” were also enriched in phenylpropanoid biosynthesis and phenylalanine metabolism (**Figure 8E**), which is consistent with the results of the metabolome. This result suggests that phenolic compounds were generally induced by the exposure of the fruit to VOCs.

Phenolic compound biosynthesis pathways mainly include the shikimate pathway, chorismic acid pathway, and phenylalanine pathway. In this study, multiple genes involved in shikimate biosynthesis and phenylalanine metabolism were markedly upregulated in the VOCs-treated group (**Figure 8G**; **Supplementary Table 3**). In the shikimate pathway, the genes (*Solyc01g105380.2* and *Solyc04g074480.3*) encoding DAHPS, the gene (*Solyc06g084460.4*) encoding SDH, the gene (*Solyc04g049350.4*) encoding CS, the gene (*Solyc06g071030.3*) encoding ICS, and the gene (*Solyc06g074530.1* and *Solyc11g066890.1*) encoding ADT were upregulated in the BF vs. CK comparison. The gene (*Solyc02g094420.3*) encoding SK exhibited no significant differences in the BF vs. CK comparison but was downregulated in the F0 vs. CK comparison. In the phenylalanine metabolism pathway, the genes (*Solyc09g007920.4* and *Solyc09g007900.5*) encoding PAL, the gene (*Solyc05g047530.3*) encoding C4H, the gene (*Solyc12g042460.2*) encoding 4CL, the genes (*Solyc11g071470.1*, *Solyc01g008300.2*, *Solyc05g039950.2* and *Solyc05g014330.1*) encoding HCT, the gene (*Solyc10g078220.2*) encoding p-coumaroyl quinate/shikimate 3′-hydroxylase (C3’H), and the gene (*Solyc02g084570.5*) encoding ferulate-5-hydroxylase (F5H) were upregulated in the BF vs. CK and F0 vs. CK comparisons. Notably, the expression levels of these genes were always more substantially upregulated in the BF vs. CK group than in the F0 vs. CK group.

## 4 Discussion

**Figure 9:**
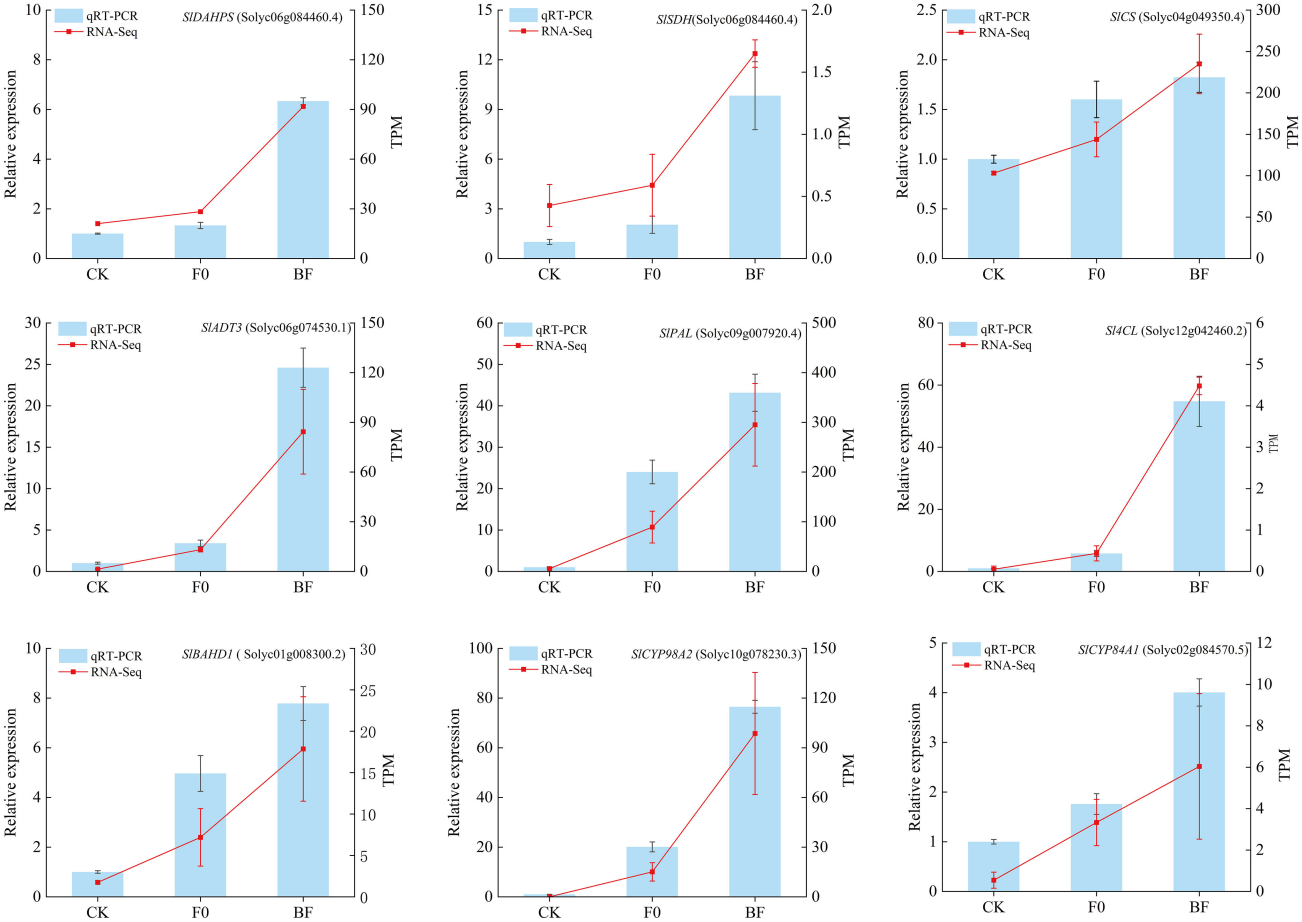
qRT-PCR validation of core genes associated with shikimate pathway and phenylalanine metabolism pathway in tomato fruit tissue. The qRT-PCR expression levels were calculated as a ratio relative to the level of expression in the CK, which was set as 1. Values are the mean ± standard deviation (SD) of three replicates.under *B. cinerea*.

In order to validate the accuracy and reproducibility of transcriptomic analysis in this study, qRT-PCR was performed on a group of DEGs related to shikimate pathway and phenylalanine metabolism pathway. **Figure 9** presented the expression levels of all 9 selected DEGs determined by qRT-PCR and RNA-Seq. The core genes of shikimate pathway, namely *SlDHQS*, *SlSDH*, *SlCS*, and *SlADT3*, as well as the key genes of phenylalanine metabolism pathway, including *SlPLA*, *Sl4CL*, *SlBAHD1*, *SlCYP98A2* and *SlCAP84A1*, exhibited higher expression in fruit tissues induced with VOCs induced treatment (BF group) compared to non-induced fruit tissues (F0 group) responding to *B. cinerea* infection. Most genes showed similar expression patterns using both methods, indicating that the expression data obtained through RNA-Seq in this study were reliable.

Microbial VOCs have been recognised as efficient chemical exchange media that mediate interchanges and defence between species (Thorn and Greenman, 2012). For example, in microbe-plant interactions, VOCs serve as key signalling molecules for regulating certain physiological responses in plants, including plant growth promotion and plant resistance induction (Poveda, 2021). Moreover, because of their excellent antifungal potential, VOCs are important alternatives to synthetic fungicides and have received increasing attention owing to their ability to reduce decay (Mari et al., 2016). Our earlier studies confirmed the direct suppression of grey mould and the maintenance of fruit quality by fumigating postharvest tomatoes with VOCs from the bacterium ETR-B22 (Chen et al., 2022). In this study, we found that fumigation with VOCs on tomato fruits was effective in reducing the disease development of *B. cinerea*. A marked reduction in disease incidence and lesion diameter observed after VOCs treatment might be attributable to its ability to enhance the internal resistance of tomato fruits, rather than to a direct fungicidal activity. Because the bacterial VOCs were removed after the fumigation as well as the tomato fruits were moved into a new sterile container, little or no VOCs remained on the skin of the fruit, excluding any direct interaction with pathogens. Combined with the results of our previous characterisation of VOCs emitted by ETR-B22 (Chen et al., 2020), we inferred that dimethyl trisulfide, methyl salicylate, and methyl benzoate may be specific inducible compounds (Naznin et al., 2014; Park et al., 2009). The incidence of grey mould in tomato fruits induced by VOCs was higher than that induced by direct fumigation in terms of incidence and spot size. This may be related to the characteristics of the VOCs mixture or the lower concentration of substances that exerted an inducing effect. In general, our findings contribute to the development of a novel resistance-inducing agent to enhance resistance against *B. cinerea* and reduce economic losses of postharvest tomato fruits.

Defence-related enzymes are closely related to the resistance of plants to pathogen infection, and the current study also indicates that the activation of coordinated defence-related enzymes is an early event in induced resistance in plants. Under biotic stress, ROS proliferate, which serves not only as a protectant against invading pathogens but also as a significant signal that activates further plant defence reactions (Lehmann et al., 2015). However, excessive accumulation of ROS leads to oxidative damage to nucleic acids, proteins, and lipids, among others, in plant cells. Enzymatic ROS-scavenging mechanisms in plants rely on antioxidant enzymes, including glutathione peroxidase, POD, PPO, SOD, APX, and CAT (Barna et al., 2003). In this study, VOCs treatment promoted higher activities of APX, POD, and PPO, as well as their gene expression after infection, possibly resulting in the regulation of ROS in tomato fruits to reduce oxidative damage (Wang et al., 2019). Moreover, the rapid accumulation of PPO and POD at significant levels at an early stage in VOCs-treated fruits may contribute to lignin formation to strengthen the plant cell walls (Zhao et al., 2016). CHI and GLU have been widely used in the defence against pathogen attacks by degrading the fungal cell wall and disrupting its structure (van Loon et al., 2006). We found that the activity and expression of CHI and GLU were markedly enhanced in VOCs-treated fruits than those in the control. A similar phenomenon was observed in other harvested fruits treated with resistance-inducing elicitors (Sun et al., 2024). Following pathogenic infections, plants synthesise secondary metabolites to resist further disease development. PAL is the first rate-limiting enzyme in the phenylpropanoid metabolic pathway and is responsible for phenol and flavonoid biosynthesis (Shadle et al., 2003). In the current study, the dramatic accumulation of PAL activity and the induction of mRNA levels were mostly found as a result of postharvest VOCs treatment in tomato fruits, indicating that VOCs treatment enhanced the disease resistance of tomato fruits, which may be attributed to the biosynthesis of defence-related secondary metabolites.

Plant defence responses against pathogens can be transferred by activating different hormone signalling pathways, thus establishing IR (Bürger and Chory, 2019) This study showed that the levels of the SA and JA pathway genes involved in biosynthesis and transduction were upregulated in the VOCs-treated group, whereas the expression of ethylene receptor genes and ethylene response factors were markedly inhibited (**Figure 5**), suggesting that the SA and JA signalling pathways may participate in VOCs-induced resistance in tomato fruits against *B. cinerea* attack, independent of the ET pathway. This result is similar to the observations of Sharifi and Ryu (2016) They found that the VOCs released by *Bacillus subtilis* GB03 mediated ISR in *Arabidopsis* mainly through the activation of SA and JA signal transduction. However, Nie et al. (2017) showed that VOCs produced by *Bacillus cereus* AR156 trigger a defence response against *B. cinerea* via JA/ET/NPR1-dependent pathways. Ryu et al. (2004) demonstrated that VOC-mediated ISR induced by *Bacillus pumilus* T4 against *Pseudomonas syringae* was ET-dependent but SA, JA, and NPR-independent. In conclusion, microbial VOCs activate plant resistance by forming a complex crosstalk with diverse signalling networks, which may be closely related to host specificity, pathogen types, and volatile profiles. Notably, genes in the JA pathway were downregulated to a different extent in VOC-treated fruits at the early stage of pathogen attraction, indicating that there is a possible antagonistic regulatory relationship between SA and JA; higher SA levels suppress JA signalling (Robert-Seilaniantz et al., 2011).

Phenolic acids play an integral role in plant immunity against various pathogens such as bacteria, fungi, and viruses. The concentration of these phenolics at infection sites, triggered by pathogen invasion, not only fortifies the cell wall structure but also aids in the reduction of reactive oxygen species, thereby expediting cross-linking reactions within the cell wall and enhancing disease resistance (Mnich et al., 2020; Zhang et al., 2020). For instance, postharvest resistance in citrus, triggered by *Pichia galeiformis*, is attributed to substantially elevated levels of phenolic acids (Chen et al., 2021). Li et al. (2021) found that 4-carvomenthenol incites resistance to *B. cinerea* in ‘Hongyan’ strawberries by promoting the biosynthesis of phenylpropanes through their metabolomics analysis, thereby accumulating phenolic acids. In our metabolomic analysis, the samples showed heightened phenolic acid enrichment following fruit induction by VOCs. Of the 51 phenolic acids that exhibited significant changes, 39 showed a notable increase (**Figure 6**). The key intermediates related to phenylalanine metabolism, including cinnamic acid, sinapic acid, ferulic acid, p-coumaryl alcohol, sinapinaldehyde, and p-coumaraldehyde, were markedly more abundant in the VOC-treated samples than in the untreated samples (**Figure 7**). These key intermediates demonstrate substantial *in vitro* antifungal activity. Recent studies have further highlighted that host resistance can also be improved by the activation of the phenylpropane pathway using exogenous ferulic and p-coumaric acids (Liu et al., 2020; Shu et al., 2021). This suggests that the significant enrichment of these critical intermediates in the phenylalanine metabolism pathway may enhance postharvest grey mould resistance in VOCs-treated tomatoes. Furthermore, we identified that modification products of the mentioned intermediates, primarily consisting of two types of phenolic acids, hydroxycinnamic acid and hydroxybenzoic acid, namely chlorogenic acid, isochlorogenic acid A, 4-hydroxybenzoic acid, 2,5-dihydroxybenzoic acid, 2,5-dihydroxybenzaldehyde, 5-O-p-coumaroylquinic acid, 3-O-p-coumaroylquinic acid, 5-O-p-Coumaroylshikimic acid O-glucoside, 2-hydroxycinnamic acid, and α-hydroxycinnamic acid, considerably accumulated after exposure to VOCs. This may have contributed to the improvement in both antioxidant and antimicrobial activities (Xu et al., 2022), thus enhancing tomato defence against *B. cinerea*. For instance, current research identifies hydroxycinnamic acid and its derivatives as a critical class of secondary metabolites recognised as biomarkers for assessing plant resistance (Liu et al., 2022). Chlorogenic acids, within the hydroxycinnamic acid derivative family, are synthesised through the esterification of caffeic and quinic acids. Their extensively studied antioxidant properties make them promising bioactive compounds (Jiao et al., 2018). It has been reported that more resistant plant species have been reported to have higher levels of chlorogenic acid concentration (Li et al., 2021). These results suggest that VOCs exposure-specific synthesis of phenolic acid components enhances fruit resistance to grey mould.

Although metabolomics can reveal what is happening, transcriptomics can provide a full view of what is happening in the cells. In the transcriptomic analysis, we compared VOCs-induced and untreated fruits after inoculation with grey mould with healthy fruits, where DEGs were significantly enriched in the phenylpropanoid biosynthesis and phenylalanine metabolism(**Figure 8D**), suggesting that the two most prominent pathways related to plant secondary metabolite biosynthesis are prevalent in *B. cinerea.* These two pathways are key to phenolic acid biosynthesis (Ma et al., 2016). In this case, shikimate pathway, as an upstream process of phenolic acid metabolism, exerts an irreplaceable impact on enhancing plant resistance. The shikimate pathway started with phosphoenolpyruvate (PEP) and erythrose-4-phosphate, which was catalysed by 3-Deoxy-D-arabino-heptulosonate-7-phosphate synthase (DAHPS) 3-dehydroquinate synthase (DHQS), shikimate dehydrogenase (SDH), shikimate kinase (SK), chorismate synthase (CS), and arogenate dehydratase (ADT), resulting in the production of phenylalanine. Activating the phenylalanine metabolism pathway lead to the synthesis of phenolic acids and other resistance metabolites, thereby enhancing postharvest fruit resistance (Yokoyama et al., 2021). PAL, Cinnamate-4-Hydroxylase (C4H), 4-Coumarate Ligase (4CL), F5H, and Caffeic Acid O-Methyltransferase are the major enzymes involved in the phenylalanine metabolic pathway, which lead to the production of different phenolic acid compounds. CAD, CCR, and UGT72E mediate the synthesis of different phenolic acid derivatives. HCT, 4CL, and E2.1.1.104 produce various phenolic acid compounds (Lin et al., 2020). The upregulation of genes encoding enzymes involved in shikimate pathway and phenylalanine metabolism pathway typically induces enhanced accumulation of phenolic acids in plants. Based on the expression profiles potentially involved in these two pathways obtained through qRT-PCR and RNA-Seq analyses, the expression levels of nine key genes (*SlDAHPS*, *SlSDH*, *SlCS*, *SlADT3, SlPAL*, *Sl4CL*, *SlBAHD1*, *SlCYP98A2* and *SlCAP84A1*) which related to the phenolic acid biosynthesis were found to be increased in both VOCs-induced and non-induced treatments after *B. cinerea* infection compared to healthy tomato fruits (**Figure 9**). However, the increase trend of all genes in VOCs-treated fruit was higher than that in the non-treated fruit, indicating that the phenolic acid metabolism in the tomato tissues induced by VOCs responded more intensively than in the non-treated group. Similar previous studies have also reported that the genes of phenolic acids biosynthesis could be significantly activated, participating to plant resistance, whether it was innate immune or induced resistance, such as disease resistance of high resistant variety of walnut (Xu et al., 2022), hot air-treated tomato (Wei et al., 2017), fulvic acid-induced table grape (Xu et al., 2019), and sulfide-treated kiwifruit (Duan et al., 2022). Overall, these results demonstrated that VOC-induced disease resistance was linked to metabolites and gene expression levels associated with phenolic acid metabolism pathway in tomato fruit.

## 5 Conclusions

**Figure 10:**
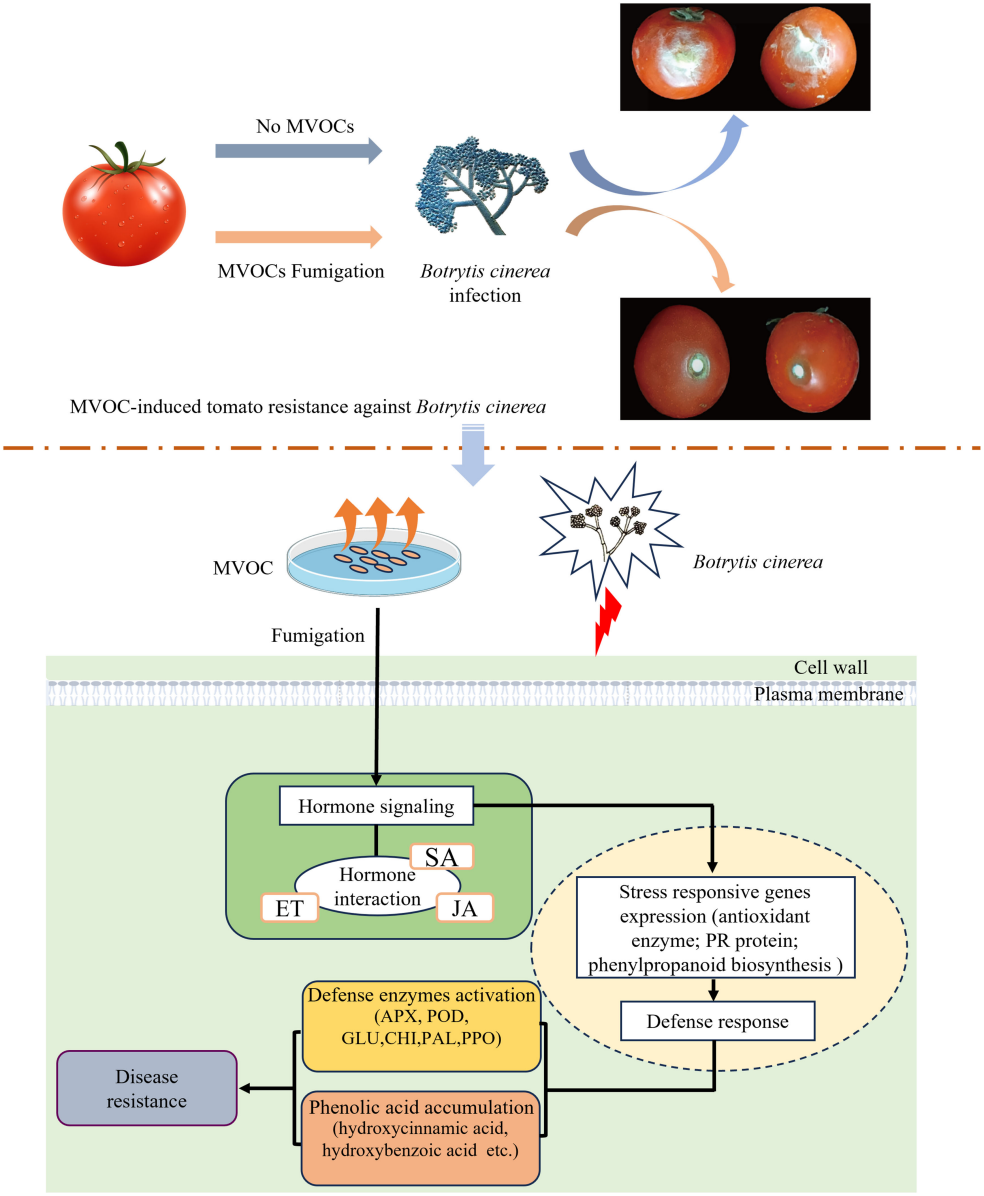
The response patterns of VOCs treatment in tomato fruits infection. Bacterial VOCs activated defense-related enzymes, regulated hormone-related genes, upregulated expression of phenolic acid synthesis genes and promoted the production of phenolic acids.

In summary, the exposure of tomato fruits to bacterial VOCs induced resistance to grey mould and reduced postharvest fruit rot. This may be related to the ability of VOCs to activate defence-related enzymes, induce SA and JA biosynthesis and signal transduction of plant hormones, and upregulate key genes. Furthermore, we investigated VOCs-induced transcriptomic and metabolomic responses to *B. cinerea* infection in fruit tissues and discovered that VOCs regulate the production of phenolic acids by upregulating the expression of genes in the shikimate pathway and that phenylalanine metabolism is involved in disease resistance enhancement (**Figure 10**). Our findings revealed the VOCs-mediated defence response mechanism in tomato postharvest disease control may guide the efficient utilisation of VOCs as potential resistance-inducing agents for postharvest fruits.

## Data Availability

The original contributions presented in the study are publicly available. This data can be found here: https://www.ncbi.nlm.nih.gov/, the project number is PRJNA1186667.
