# Supplementary material for: Investigating the action model of the resistance enhancement induced by bacterial volatile organic compounds against Botrytis cinerea in tomato fruit
Source: Front Plant Sci. 2024 Nov 26;15:1475416. doi: 10.3389/fpls.2024.1475416 (PMC11628293; doi:10.3389/fpls.2024.1475416)
Supplement: Supplementary file 1 [file DataSheet1.docx]

Supplementary Material

# Supplementary Table

**Supplementary** **Table 1** Primers used for qRT-PCR.

| Gene name | NCBI Accession number | Primer sequence (5’-3’) |
| --- | --- | --- |
| *Actin* | AB199316.1 | F: CTCGAGCAGTGTTTCCCAGT |
|  |  | R: GGTGCCTCAGTCAGGAGAAC |
| *PR3a* | Z15141.1 | F: TTGCTGCCTTTTTCGGTC |
|  |  | R: TGGATAGGTCCTCTGCCATAG |
| *PR2a* | M80608.1 | F: TACAAAGCAATCGGTGAAGC |
|  |  | R: GAGCATACGGAAGTGAAATCTG |
| *APX* | NM_001247859.2 | F: TCTCCAGCTGGTACTTTGATACT |
|  |  | R: TCGCAATTTTGCTGCACTGT |
| *POD3* | NM_001302921.2 | F:GAATGTCCGGGAGTTGTTTCT |
|  |  | R:TCTTCTACCAGTTGGCACATTC |
| *PAL* | NM_001320609.1 | F:CTTTGATGCAGAAGCTGAGACA |
|  |  | R:TCGTCCTCGAAAGCTACAATCT |
| *PPO* | XM_004245981.4 | F: GGAAGGCAACTGGAGGTAAA |
|  |  | R:CCAAACAGTCTCTGACTCTCAC |
| *ICS* | DQ984132.1 | F:GCAGCTTTCCTCCGTTCTTA |
|  |  | R:ATGGTCCCAAGACGCTTTAC |
| *NPR1* | KX198701.1 | F:TTGCACAAGTTGATGGCACG |
|  |  | R:TCCACTGTTGTCCTCTGTGC |
| *TGA1* | XM_004236682.4 | F: AATTGGCATGTGGGATGATTTC |
|  |  | R:GTATCTGGTCCTCTAGGCATTTC |
| *TGA2.2* | NM_001309871.1 | F:ATTCTGGTTCTCGCACTGATAC |
|  |  | R:CTGCAATAGCCCTGGATTGA |
| *PR1* | Y08804.1 | F:TGTCCGAGAGGCCAAGCTA |
|  |  | R:TGTCCGATCCAGTTGCCTA |
| *PR5* | NM_001247422.3 | F:CGGTGATTGTGGTGGAGTCT |
|  |  | R:CCTCCGGGTACCCTAAGTGA |
| *AOC* | AF384374.1 | F:GTGGAAGCCCTGCTTATCTT |
|  |  | R:TGCAGAGTCCTGCTGTTATTC |
| *AOS* | NM_001287778.2 | F:CCTCCGGTAGCTTCACAATAC |
|  |  | R:GGTTGGTACCCGAATAGGATTT |
| *COI* | NM_001247535.1 | F:CGCGGTATCGTTGGTATGTAAG |
|  |  | R:CTGCTCTGGTTTCGCTGTATAA |
| *MYC2* | NM_001324483.1 | F:TAGTAGTAGAGTGGTAGAGCCTG |
|  |  | R:ATTCAATGGCTCCTCCCGTC |
| *ETR2* | NM_001247224.2 | F: GGTAACGAAGAGTCTGCTAGTG |
|  |  | R:TACTTTGTGTTCCCGAGTAAGG |
| *ETR3* | NM_001246965.2 | F:ACTCTCTTAAACGTGGCGGG |
|  |  | R:CTGGTTTGGCAACTGAAGCC |
| *ETR4* | AF118843.1 | F:GATCAGGTTGCTGTGGCTCT |
|  |  | R:CCTTGCTTGACTCGCCCTAA |
| *ERF1* | NM_001247912.2 | F:GGTCCTTGGTCTCTACTCAATTT |
|  |  | R: AACAGCAGCTGGAGATAATCC |

**Supplementary** **Table 2** Differentially accumulated phenolic acids from widely targeted metabolomic profiling of BF vs. CK group.

| Compounds of phenolic acid | VIP | P-value | Log_2_(Fold change) | Type |
| --- | --- | --- | --- | --- |
| 5-O-p-Coumaroylshikimic acid O-glucoside | 1.18 | 6.07E-03 | 1.09 | up |
| 4-Hydroxybenzoic acid | 1.19 | 1.60E-03 | 2.32 | up |
| Methyl salicylate-2-O-glucoside | 1.13 | 4.51E-02 | 1.21 | up |
| 5-O-p-Coumaroylquinic acid | 1.20 | 1.07E-04 | 2.39 | up |
| 4-Aminobenzoic acid | 1.18 | 9.63E-03 | 2.97 | up |
| Isochlorogenic acid B | 1.17 | 1.57E-04 | 1.88 | up |
| 2-Hydroxycinnamic acid | 1.19 | 2.81E-04 | 1.85 | up |
| α-Hydroxycinnamic acid | 1.19 | 3.52E-03 | 2.15 | up |
| Chicoric Acid | 1.18 | 4.26E-04 | 1.25 | up |
| Sinapic acid | 1.18 | 1.46E-02 | 1.17 | up |
| Cinnamic acid | 1.19 | 8.21E-03 | 1.98 | up |
| 4-Hydroxybenzaldehyde | 1.19 | 2.73E-04 | 1.33 | up |
| 2,5-Dihydroxybenzaldehyde | 1.19 | 8.58E-05 | 2.28 | up |
| 5-O-Caffeoylshikimic acid | 1.19 | 3.27E-05 | 1.42 | up |
| p-Coumaryl alcohol | 1.19 | 7.54E-04 | 4.35 | up |
| Chlorogenic acid | 1.17 | 3.44E-02 | 1.81 | up |
| 3-O-p-Coumaroylquinic acid | 1.19 | 2.73E-04 | 2.22 | up |
| P-Methoyxcinnamate glucoside | 1.18 | 9.85E-04 | 1.84 | up |
| p-Coumaric acid ethyl ester | 1.20 | 5.91E-03 | 11.46 | up |
| Isochlorogenic acid A | 1.19 | 5.18E-03 | 1.11 | up |
| Benzoic acid | 1.12 | 8.01E-03 | 1.48 | up |
| 2,5-Dihydroxybenzoic acid | 1.19 | 9.50E-05 | 2.02 | up |
| Sinapinaldehyde | 1.11 | 3.93E-03 | 1.13 | up |
| Protocatechuic acid methyl ester | 1.19 | 3.62E-03 | 3.88 | up |
| 4-Coumaroylshikimate | 1.17 | 1.45E-02 | 2.41 | up |
| Dicaffeoylquinic acid-O-glucoside | 1.14 | 2.74E-02 | 1.17 | up |
| 4-(3,4,5-Trihydroxybenzoxy)benzoic acid | 1.20 | 1.75E-02 | 11.83 | up |
| p-Coumaraldehyde | 1.14 | 4.84E-04 | 2.37 | up |
| 3-Hydroxycinnamic acid | 1.19 | 7.33E-04 | 2.03 | up |
| 2-Methoxy-4-ethenylphenol | 1.20 | 8.98E-04 | 2.29 | up |
| 3,4-Dimethoxycinnamic acid | 1.19 | 1.93E-03 | 3.09 | up |
| Ferulic acid | 1.19 | 3.68E-05 | 2.73 | up |
| Dihydroferulic acid | 1.19 | 4.13E-03 | 1.15 | up |
| 4-Hydroxycinnamic acid p-hydroxyphenethylamine | 1.20 | 1.64E-03 | 3.94 | up |
| Ethyl ferulate | 1.18 | 2.24E-03 | 1.23 | up |
| Trans-5-O-(p-Coumaroyl)shikimate | 1.16 | 1.45E-02 | 2.41 | up |
| 6-O-Feruloyl-β-D-glucose | 1.19 | 1.63E-04 | 1.53 | up |
| 1-O-Feruloylquinic acid | 1.19 | 2.74E-02 | 11.84 | up |
| Vanillic acid-4-O-glucoside | 1.18 | 2.08E-03 | 1.20 | up |
| 2-(Formylamino)benzoic acid | 1.19 | 1.02E-04 | -1.05 | down |
| Ferulic acid methyl ester | 1.19 | 4.13E-03 | -7.72 | down |
| Calceolarioside D | 1.19 | 6.33E-03 | -2.30 | down |
| Phenyl acetate | 1.20 | 6.01E-04 | -3.59 | down |
| 4-Hydroxyphenylacetic acid | 1.20 | 5.30E-04 | -5.42 | down |
| 4-Hydroxyacetophenone | 1.20 | 1.01E-05 | -3.57 | down |
| Citrusin B | 1.20 | 1.91E-02 | -10.45 | down |
| Mandelic acid-β-glucoside | 1.14 | 2.87E-03 | -1.28 | down |
| 4-Hydroxyphenylethanol | 1.19 | 1.45E-02 | -2.60 | down |
| P-Methoyxcinnamate glucoside | 1.18 | 9.85E-04 | -1.84 | down |
| p-hydroxyphenethylamine | 1.20 | 1.64E-03 | -3.94 | down |
| Feruloylcaffeoyltartaric acid | 1.13 | 7.76E-04 | -2.91 | down |
| Syringic acid | 1.09 | 4.24E-02 | -1.37 | down |
| 3-hydroxyphenylacetic acid | 1.20 | 5.74E-04 | -5.44 | down |
| Grevilloside F | 1.19 | 5.10E-04 | -1.67 | down |
| 4-Hydroxyphenyllactic acid | 1.18 | 4.20E-03 | -1.43 | down |
| 2',4',6'-Trihydroxyacetophenone | 1.04 | 8.92E-03 | -1.44 | down |
| 2-(Formylamino)benzoic acid | 1.19 | 1.02E-04 | -1.05 | down |
| 4-Hydroxy-3,5-diisopropylbenzaldehyde | 1.20 | 1.66E-04 | -9.25 | down |
| Methyl 3-(3-hydroxy-4-methoxyphenyl)  propanoate | 1.19 | 9.36E-03 | -2.31 | down |
| 5-Glucosyloxy-2-Hydroxybenzoic acid methyl ester | 1.20 | 2.09E-03 | -13.64 | down |
| Methyl 2,4-dihydroxyphenylacetate | 1.15 | 9.25E-04 | -1.62 | down |

**Supplementary** **Table S3** The detailed information of DEGs in phenylalanine upstream metabolism and phenolic acids biosynthesis in the BF vs. CK and F0 vs. CK comparison groups.

| Pathway | Enzyme name | Definition (EC) | Gene ID | Regulated  (Log_2_FoldChange) | |
| --- | --- | --- | --- | --- | --- |
|  |  |  |  | F0vsCK | BFvsCK |
| Shikimate pathway | DAHPS | 3-deoxy-d-arabino-heptulosonate 7-phosphate synthase [EC:2.5.1.54] | Solyc01g105380.2 | 0.8231 | 5.2244 |
|  |  |  | Solyc04g074480.3 | 0.43 | 2.47265 |
|  | SDH | shikimate dehydrogenase [EC:4.2.1.10] | Solyc06g084460.4 | 0.97 | 2.86 |
|  | CS | chorismate synthase [EC:4.2.3.5] | Solyc04g049350.4 | 0.51 | 1.18 |
|  | ICS | isochorismate synthase [EC:5.4.4.2] | Solyc06g071030.3 | 0.77 | 1.51 |
|  | ADT | arogenate dehydratase | Solyc06g074530.1 | 3.78 | 6.30 |
|  |  |  | Solyc11g066890.1 | -0.45 | 1.41 |
| Phenylalanine pathway | PAL | phenylalanine ammonia-lyase [EC:4.3.1.24] | Solyc09g007920.4 | 4.51 | 6.19 |
|  |  |  | Solyc09g007900.5 | 3.29 | 4.99 |
|  | CYP73A | Cinnamate 4-hydroxylase [EC:1.14.14.91] | Solyc05g047530.3 | 6.41 | 5.77 |
|  | 4CL | 4-coumarate-CoA ligase [EC:6.2.1.12] | Solyc12g042460.2 | 3.44 | 6.68 |
|  | HCT | Hydroxycinnamoyl transferase [EC:2.3.1.133] | Solyc11g071470.1 | 5.11 | 8.83 |
|  |  |  | Solyc01g008300.2 | 2.41 | 3.61 |
|  |  |  | Solyc05g039950.2 | 3.15 | 4.68 |
|  |  |  | Solyc05g014330.1 | 5.13 | 5.16 |
|  | C3’H | p-coumaroyl quinate/shikimate 3′-hydroxylase [EC:1.14.14.96] | Solyc10g078220.2 | 9.10 | 11.34 |
|  |  |  | Solyc10g078230.3 | 8.46 | 11.04 |
|  | F5H | Ferulate-5-hydroxylase  [EC:1.14.-.-] | Solyc02g084570.5 | 3.74 | 3.88 |

Note: CK: healthy tomato fruits without any treatment; BF: Inoculated fruit with VOCs induction; F0: Inoculated fruit without VOCs induction.

**Table S4** Primers used for qRT-PCR in transcriptomic analysis

| Gene name | Gene ID | Primer sequence (5’-3’) |
| --- | --- | --- |
| *Actin* | AB199316.1 | F: CTCGAGCAGTGTTTCCCAGT |
|  |  | R: GGTGCCTCAGTCAGGAGAAC |
| *SlDAHPS* | Solyc04g074480.3 | F: TAACACGCCGGGATTCAACT |
|  |  | R: TCAACATGGGCACCATCCAA |
| *SlSDH* | Solyc06g084460.4 | F: AAGCAGCAGTACGGTGTTGT |
|  |  | R: TCAATTGCCGTCACACAAGC |
| *SlCS* | Solyc04g049350.4 | F: GGATGAGCATGGCCGAATCA |
|  |  | R: CCACACAAGGATCATGGCGA |
| *SlADT* | Solyc11g066890.1 | F: CCTCGAACCGATCGTCCATT |
|  |  | R: TACTCGAAATGCTTCGCCGT |
| *SLPAL* | Solyc09g007920.4 | F: CCGGTGAGGAGATCGACAAG |
|  |  | R: GGAGCACCATTCCAGCTCTT |
| *Sl4CL* | Solyc12g042460.2 | F: AGTCATTTCTGGGGCAGCAC |
|  |  | R: GTGCCACATGCCCCTGATTT |
| *SlAHT1* | Solyc11g071470.1 | F: GGCCACCACATGATAGCTGA |
|  |  | R: AGGGCGGAAAATAGCACGAT |
| *SlCYP98A2* | Solyc10g078230.3 | F: GAATGCACCCTCCAACTCCA |
|  |  | R: GACCTAACGCCCACACGTT |
| *SlCYP84A1* | Solyc02g084570.5 | F: AATTGGCATGTGGGATGATTTC |
|  |  | R: TCTCCGACTTCACCCTCCAA |
|  |  | R: AGTAGCCCGAGACTGTCGAT |

# Supplementary Figures


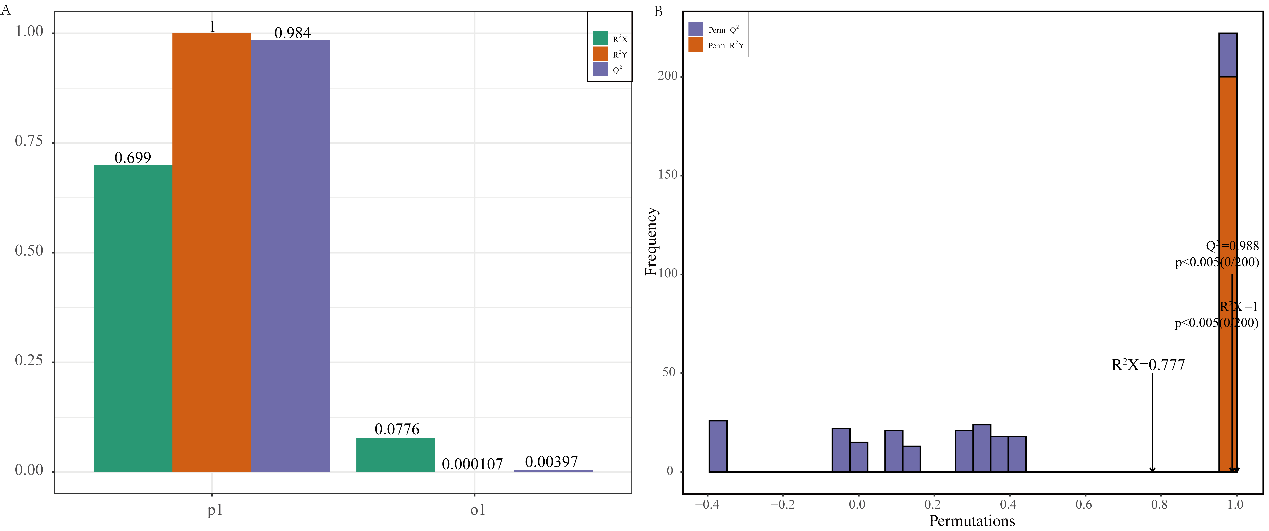


**Supplementary Figure 1.** Model verification of OPLS-DA. (A) OPLS-DA model profiles for BF vs F0. (B) Permutation test of the OPLS-DA model between the CK vs F0. High predictability (Q2) and strong goodness of fit (R2X, R2Y) of the OPLS-DA models were observed in the comparison between BF vs F0 (Q2 = 0.984, R2X = 0.777, R2Y = 1; Fig. S2A). The goodness of fit and predictive capability of the three original models are much higher compared to ratios based on the permutation model (P < 0.005). BF: Inoculated fruit with VOCs induction; F0: Inoculated fruit without VOCs induction.
